# Independent and joint cross-sectional associations of statin and metformin use with mammographic breast density

**DOI:** 10.1186/s13058-020-01336-0

**Published:** 2020-09-15

**Authors:** Erica J. Lee Argov, Teofilia Acheampong, Mary Beth Terry, Carmen B. Rodriguez, Mariangela Agovino, Ying Wei, Shweta Athilat, Parisa Tehranifar

**Affiliations:** 1 Department of Epidemiology, Columbia University Mailman School of Public Health, 722 W 168th St, New York, NY USA; 2 Department of Biostatistics, Columbia University Mailman School of Public Health, 722 W 168th St, New York, NY USA

**Keywords:** Breast density, Metformin, Mammography, Hydroxymethylglutaryl-CoA reductase inhibitors, Breast neoplasms, Anticarcinogenic agents

## Abstract

**Background:**

Well-tolerated and commonly used medications are increasingly assessed for reducing breast cancer risk. These include metformin and statins, both linked to reduced hormone availability and cell proliferation or growth and sometimes prescribed concurrently. We investigated independent and joint associations of these medications with mammographic breast density (MBD), a useful biomarker for the effect of chemopreventive agents on breast cancer risk.

**Methods:**

Using data from a cross-sectional study of 770 women (78% Hispanic, aged 40–61 years, in a mammography cohort with high cardiometabolic burden), we examined the association of self-reported “ever” use of statins and metformin with MBD measured via clinical Breast Imaging Reporting and Data System (BI-RADS) density classifications (relative risk regression) and continuous semi-automated percent and size of dense area (Cumulus) (linear regression), adjusted for age, body mass index, education, race, menopausal status, age at first birth, and insulin use.

**Results:**

We observed high statin (27%), metformin (13%), and combination (9%) use, and most participants were overweight/obese (83%) and parous (87%). Statin use was associated with a lower likelihood of high density BI-RADS (RR = 0.60, 95% CI = 0.45 to 0.80), percent dense area (PD) (*β* = − 6.56, 95% CI = − 9.05 to − 4.06), and dense area (DA) (*β* = − 9.05, 95% CI = − 14.89 to − 3.22). Metformin use was associated with lower PD and higher non-dense area (NDA), but associations were attenuated by co-medication with statins. Compared to non-use of either medication, statin use alone or with metformin were associated with lower PD and DA (e.g., *β* = − 6.86, 95% CI: − 9.67, − 4.05 and *β* = − 7.07, 95% CI: − 10.97, − 3.17, respectively, for PD) and higher NDA (*β* = 25.05, 95% CI: 14.06, 36.03; *β* = 29.76, 95% CI: 14.55, 44.96, respectively).

**Conclusions:**

Statin use was consistently associated with lower MBD, measured both through clinical radiologist assessment and continuous relative and absolute measures, including dense area. Metformin use was associated with lower PD and higher NDA, but this may be driven by co-medication with statins. These results support that statins may lower MBD but need confirmation with prospective and clinical data to distinguish the results of medication use from that of disease.

## Introduction

Risk of incident and recurrent breast cancer may be reduced by agents such as selective estrogen receptor modulators and aromatase inhibitors; however, broad adverse effects limit their uptake and acceptability [1, 2]. Well-tolerated, commonly used medications for multiple chronic conditions with potential for reducing cancer risk may offer more widespread and sustained use [3–6]. Metformin, a biguanide used to treat type 2 diabetes, has antiproliferative effects, has been linked to reduced cell growth and circulating estrogen, and has been recommended to reduce the risk of breast cancer recurrence though the evidence is less consistent for incidence [7–13]. The evidence for statins, HMG-CoA reductase inhibitors used to treat high cholesterol that may reduce the cholesterol available for hormone synthesis and cell proliferation, is stronger but similarly more consistent for recurrence than incidence, and particularly congruous for lipophilic statins [7, 14–19]. Little is known about the combined use of these drugs and breast cancer risk, though combined use can be common in individuals with type 2 diabetes who are at higher risk for cardiovascular disease [20].

Hypothesized mechanisms for these medications’ effects on breast cancer—potentially involving hormone synthesis, cell growth, and proliferation—may also affect mammographic breast density (MBD), a useful biomarker for the effect of chemopreventive agents on breast cancer. For example, metformin’s AMPK-dependent effects inhibit aromatase, thereby inhibiting androgen to estrogen conversion, and statins inhibit cholesterols—necessary for estrogen synthesis—along the mevalonate pathway, which is also dysregulated in certain breast cancer subtypes [4, 5, 21–23]. Studies assessing statin/metformin use and MBD in trials or populations with comparatively low rates of use of either medication have been inconsistent [24–31]. We aimed to investigate the independent and joint associations of metformin and statins, often prescribed concurrently, use with MBD in a population with a high prevalence of cardiometabolic risk factors and therefore having a high prevalence of medication use.

## Methods

We evaluated the association between medication use and MBD in the New York Mammographic Density (NY MaDe) study, an ongoing screening cohort of women, ages 40–60 years at recruitment from mammography screening appointments at a New York City facility from 2016 through 2018. This cohort has been observed to have a high prevalence of cardiometabolic risk factors [26, 32]. The response rate was approximately 42%; however, the enrolled sample is socio-demographically representative of the community served by the clinic. At enrollment, we conducted in-person interviews in both English and Spanish to collect participants’ self-reported medication use, socio-demographics, breast cancer-related risk factors, and measured height and weight to calculate BMI in kilograms per meter square. We also obtained participants’ mammography clinical reports containing Breast Imaging Reporting and Data System (BI-RADS) density classifications, and digital mammograms from which we derive continuous MBD measures. We excluded 4 women with a history of breast cancer and 37 women for missing continuous MBD measures (*n* = 31), BI-RADS density categories (*n* = 1), education (*n* = 1), metformin (*n* = 3) or statin use (*n* = 1), or BMI (*n* = 4). The final analytic sample size was 770. This study received Columbia University Medical Center Institutional Review Board approval, and all participants provided written informed consent.

To obtain medication use, data we asked “Have you ever taken or are you currently taking any of the following types of medications?” For statin use, we asked if women had “Ever taken?” (Yes, No, Don't know) “Anti-cholesterol drugs called statins (e.g., Simvastatin).” For metformin use, we asked about “Metformin to treat diabetes or other conditions.” Women responding “Yes” to this question were also asked if they were “Currently taking?” (Yes, No, Don't know) that medication. Women were categorized as ever or never users, based on their answers to the first question. To examine co-medication, women were categorized into the following groups: ever used metformin and ever used statin, ever used metformin and never used statin, ever used statin and never used metformin, and never used statin and never used metformin.

Using a semi-automated thresholding method (Cumulus software (Toronto, Canada)), we derived the following continuous MBD measures from digital mammograms: centimeter square dense area (absolute dense breast tissue), centimeter square non-dense area (predominantly fatty breast tissue, total breast area – dense area), and percent density (the relative amount of dense breast tissue, dense area/total breast area × 100%). Craniocaudal images of the left breast were randomly sorted into batches of ~ 50 and were read by a blinded, trained reader (SA). We randomly duplicated 10% of a single batch across all batches and 10% of images within each batch to establish between and within batch reliability measures, respectively. Between and within batch reliability coefficients were ≥ 0.93 for all density measures.

We compared the distributions of breast cancer risk factors and socio-demographic characteristics by medication use with chi-squared or Fisher’s exact tests for categorical variables and two-sample *t* tests or ANOVA for continuous variables. We used linear regression to examine the association between medication use and continuous MBD (percent density, dense area, and non-dense area). We used Poisson regression with robust error variance to assess whether medication use was associated with high density BI-RADS (heterogeneously or extremely dense (C + D) vs. almost entirely fatty or scattered fibroglandular densities (A + B)). For each outcome, we used a multi-stage modeling strategy that sequentially included confounders to better understand the underlying confounding pattern. We first fit separate models for each medication use adjusted for only the socio-demographic factors, i.e., age at enrollment (continuous), education (high school or less, more than high school but not Bachelor’s degree, Bachelor’s or higher degrees), and race (Hispanic, Non-Hispanic White, Non-Hispanic Black, Non-Hispanic Asian, or other race). To this model, we added BMI (continuous). Then, we included the following covariates based on a combination of a priori hypotheses and differences observed in bivariate analyses: menopausal status (pre-/peri-menopausal, post-menopausal), age at first live birth (nulliparous, < 25 years old, 25 to < 35 years old, ≥ 35 years old), and insulin use (ever vs. never). Finally, we mutually adjusted for statin and metformin use (ever vs. never) to assess independent associations. Co-medication models assessing joint effects were adjusted for the same set of covariates. Sensitivity analyses were conducted to assess whether results differed if excluding insulin users, by current vs. prior users, by BMI category (BMI < 25, 25 to < 30, 30 to < 35, ≥ 35), and by menopausal status (pre- and post-menopause).

To address potential residual confounding by variables not included in our final model, we conducted sensitivity analyses using propensity scores (PS) [33]. We used logistic regression models to calculate separate propensity scores for statin and metformin use, using the set of a priori covariates described above, and any variable from bivariate analyses that showed a significant association with any of our density measures or with medication use, after adjustment for age and BMI. This included other relevant medications and metabolic conditions for which data were available. For both metformin and statin, these additional variables included diabetes (type 1, type 2, gestational diabetes only, no diagnosis), benign breast disease (ever, never), breast biopsy (ever, never), aspirin use (ever, never), anti-hypertension medication used (ever, never), age at menopause (pre-/peri-menopausal, < 45 at menopause, 45 to < 50 at menopause, > 50 at menopause), age at menarche (< 12, 12, 13, > 14), and breast cancer family history (yes, no). For metformin, diabetes was excluded from its propensity score set of predictors as the disease nearly uniformly predicted metformin use, and thus, the resulting set of propensity scores had limited overlap and increased imbalance among other variables. Women missing any of these variables were excluded from this sensitivity analysis; therefore, the resulting sample sizes were 762 (statin) and 764 (metformin). We applied the propensity scores in two ways, as previously described [33, 34]: (a) PS covariate adjustment in which we regressed our density outcomes on each medication adjusted only for the propensity score and (b) by weighing participants by the inverse of their propensity score after confirming improved balance among covariates in the weighted sample such that medication use in our weighted sample should be independent of the potential confounders used to generate the PS (inverse probability of treatment weighting; IPTW).

All statistical tests were two sided, and we considered *p* values < 0.05 significant.

## Results

**Fig. 1:**
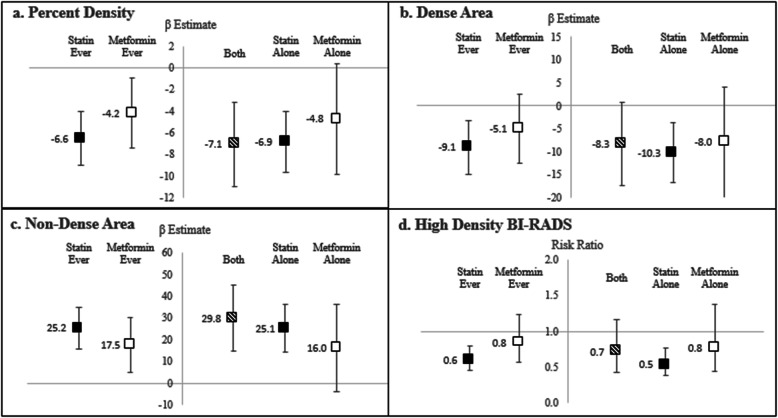
Multivariable associations of statin and metformin use and mammographic breast density. Parameter estimates and 95% confidence intervals for percent density (**a**), centimeter square dense area (**b**), centimeter square non-dense area (**c**), and high density BI-RADS (**d**) with statin and metformin use in separate fully adjusted models (left panel) as well as fully adjusted co-medication category models (right panel, reference group used neither metformin nor statin). All models adjust for age, BMI, education, race, menopausal status, age at first live birth, and insulin use

Within this study cohort, participants had a high prevalence of statin (27.1%) and metformin (13.2%) use and were mostly Hispanic (78.4%), ≥ 50 years old (64.0%), overweight or obese (82.6%), and parous (86.9%) (Table 1). Users of either medication were more likely to be older, be obese (BMI ≥ 30 kg/m^2^), have obtained less education, and have used insulin. In all multivariable models including the fully adjusted model that included BMI as well as socio-demographic and reproductive factors, ever use of statin was negatively associated with high density BI-RADS (RR = 0.60, 95% CI = 0.45 to 0.80), percent density (*β* = − 6.56, 95% CI = − 9.05 to − 4.06), and dense area (*β* = − 9.05, 95% CI = − 14.89 to − 3.22), and positively associated with non-dense area (*β* = 25.21, 95% CI = 15.47 to 34.95) (Fig. 1, Supplemental Table 1). Metformin use was not statistically significantly associated with BI-RADS density after adjustment for BMI, nor was it associated with DA, but it remained negatively associated with percent density (*β* = − 4.20, 95% CI = − 7.42 to − 0.99) and positively associated with non-dense area (*β* = 17.48, 95% CI = 4.95 to 30.00) in the fully adjusted models. When mutually adjusted, metformin’s associations with PD and NDA were largely attenuated, while statins use remained negatively associated with BI-RADS, percent density, and dense area, and positively associated with non-dense area (Supplemental Table 1).

**Table 1:** Characteristics of statin and metformin users

			Statin use					Metformin use				
	Total		Ever users		Never users			Ever users		Never users		
	***n***	%	***n***	%	***n***	%		***n***	%	***n***	%	
Total (row percent)	770	100.0	209	27.1	561	72.9		102	13.2	668	86.8	
**Medication use**												
Metformin ever	102	13.25	69	33.01	33	5.88	**	102	100.0	0	0.00	
Statin ever	209	27.14	209	100.0	0	0.00		69	67.65	140	20.96	**
Insulin ever	31	4.03	20	9.57	11	1.96	**	16	15.69	15	2.25	**
Hormone replacement therapy ever	32	4.16	10	4.78	22	3.92		5	4.90	27	4.04	
Aspirin ever	159	20.65	82	39.23	77	13.73	**	45	44.12	114	17.07	**
Anti-hypertension medications ever	294	38.18	133	63.64	161	28.70	**	69	67.65	225	33.68	**
**Demographic and medical factors**												
Age												
40 to < 45	107	13.90	8	3.83	99	17.65	**	3	2.94	104	15.57	**
45 to < 50	170	22.08	22	10.53	148	26.38		11	10.78	159	23.80	
50 to < 55	218	28.31	48	22.97	170	30.30		33	32.35	185	27.69	
55–61	275	35.71	131	62.68	144	25.67		55	53.92	220	32.93	
Mean [SD]	51.96 [5.63]		55.11 [4.64]		50.78 [5.52]		**	54.64 [4.38]		51.55 [5.69]		**
Education												
High school or less	359	46.62	114	54.55	245	43.67	*	63	61.76	296	44.31	**
Some college	179	23.25	48	22.97	131	23.35		20	19.61	159	23.80	
At least Bachelor’s	232	30.13	47	22.49	185	3298		19	18.63	213	31.89	
Race/ethnicity												
Hispanic	604	78.44	176	84.21	428	76.29	*	81	79.41	523	78.29	
Non-Hispanic white	60	7.79	8	3.83	52	9.27		2	1.96	58	8.68	
Non-Hispanic black	88	11.43	20	9.57	68	12.12		16	15.69	72	10.78	
Non-Hispanic, Asian, or other race	18	2.34	5	2.39	13	2.32		3	2.94	15	2.25	
Diabetes												
Any	160	20.78	86	41.15	74	13.19	**	94	92.16	66	9.88	**
Type 1	12	1.56	8	3.83	4	0.71	**	9	8.82	3	0.45	**
Type 2	100	12.99	68	32.54	32	5.70	**	82	80.39	18	2.69	**
Gestational	72	9.35	27	12.92	45	8.02	*	23	22.55	49	7.34	**
BMI												
18.5 to < 25	134	17.40	26	12.44	108	19.25	*	11	10.78	123	18.41	**
25 to < 30	247	32.08	62	29.67	185	32.98		19	18.63	228	34.13	
30 to < 35	215	27.92	65	31.10	150	26.74		37	36.27	178	26.65	
≥ 35	174	22.60	56	26.79	118	21.03		35	34.31	139	20.81	
Mean [SD]	30.91 [6.56]		31.80 [6.27]		30.58 [6.64]		*	33.67 [6.66]		30.49 [5.69]		**
**Reproductive history**												
Age at menarche												
< 12	204	26.49	45	21.53	159	28.34		26	25.49	178	26.65	
12	138	17.92	37	17.70	101	18.00		20	19.61	118	17.66	
13	167	21.69	52	24.88	115	20.50		26	25.49	141	21.11	
> 14	258	33.51	73	34.93	185	32.98		29	28.43	229	34.28	
Missing	3	0.39	2	0.96	1	0.18		1	0.98	2	0.30	
Parity												
Nulliparous	101	13.12	27	12.92	74	13.19		11	10.78	90	13.47	
1–2 children	368	47.79	99	47.37	269	47.95		48	47.06	320	47.90	
3 or more children	301	39.09	83	39.71	218	38.86		43	42.16	258	38.62	
Age at first live birth												
Nulliparous	101	13.12	27	12.92	74	13.19		11	10.78	90	13.47	
< 25 years old	400	51.95	114	54.55	286	50.98		63	61.76	337	50.45	
25 to < 35 years old	212	27.53	55	26.32	157	27.99		23	22.55	189	28.29	
> 35 years old	57	7.40	13	6.22	44	7.84		5	4.90	52	7.78	
**Breast cancer risk factors**												
Post-menopause	455	59.09	179	85.65	276	49.20	**	78	76.47	377	56.44	**
First-degree family history of breast cancer	95	12.34	23	11.00	72	12.83		15	14.71	80	11.98	
Age at menopause, mean [SD]	46.39 [6.30]		45.77 [6.60]		46.78 [6.09]			45.63 [6.91]		46.54 [6.18]		

Used chi-squared or Fisher’s exact test for categorical variables and two-sample *t* test for continuous; missing were excluded

*p* value comparing ever to never users denoted < 0.05 (*) or < 0.01 (**)

In our cohort, 9.0% used both metformin and statins (Supplemental Table 2). Co-medication models assessing independent effects of each medication and joint effects of co-medication showed statins used alone or in combination were negatively associated with all MBD measures in multivariable models (e.g., *β* = − 6.86, 95% CI = − 9.67 to − 4.05 and *β* = − 7.07, 95% CI = − 10.97 to − 3.17 lower percent dense area, respectively, relative to non-users of either medication). Estimates were unchanged by metformin, suggesting minimal contribution of metformin use. We observed a borderline association for metformin use alone with reduced percent density, but this association was largely attenuated after adjustment for BMI (Fig. 1, Supplemental Table 1).

Sensitivity analyses showed slightly stronger associations between statin use and dense area in current (*β* = − 11.38, 95% CI = − 18.17 to − 4.59) versus former (*β* = − 4.88, 95% CI = − 13.43 to 3.67) users relative to non-users, while the associations for percent density and non-dense area did not differ by timing of use (data not shown). Metformin use was only associated with percent density and non-dense area among current users, who comprised the majority of metformin users, but only before adjustment for statins. Excluding insulin users (*n* = 31) did not alter our results. We found no statistically significant additive interaction between either metformin or statin use and either BMI category or menopausal status, though stratified analyses suggested associations with statin use may be stronger for DA among BMI categories > 25 and for NDA among BMI categories > 30. Propensity scores used for adjustment and IPTW considered polypharmacy (e.g., aspirin use and anti-hypertension medications) and relevant metabolic conditions (e.g., benign breast disease, diabetes (only in the PS for statin use)) (Supplemental Table 2). None of the sensitivity analyses using propensity score adjustment nor IPTW materially altered statin’s effect size and associations with all density measures (Supplemental Table 3). Metformin, in its PS-adjusted model, was significantly associated with percent density, but not while adjusting for statin use. The IPTW sensitivity analysis found that metformin’s associations with percent density and non-dense area were unchanged by adjusting for statin use (Supplemental Table 3).

## Discussion

Our novel findings showed that statin use was consistently associated with lower BI-RADS, percent density, and dense area, and higher non-dense area, even when considered with metformin, use the most commonly prescribed co-medication in our cohort. Statin use in particular was consistently associated with dense area, which is less susceptible to residual confounding from adiposity and corresponds to biologically significant and breast cancer risk-relevant fibroglandular tissue where most breast tumors arise [35–39]. The associations we observed between metformin and percent density and non-dense area appeared to be attenuated by accounting for statin use, except when using the IPTW approach, where women discordant for medication use were more heavily weighted than others. Thus, although our finding may suggest that co-medication with statin may contribute to associations with density observed among metformin users, this must be cautiously interpreted given the sensitivity analysis and the small number of participants reporting only metformin use. Nevertheless, the presence and magnitude of the associations between statin use and all MBD measures in this cohort remained consistent throughout our sensitivity analyses and thus warrant future exploration in future studies.

The association between metformin use and MBD has had limited attention in the literature. Three studies evaluating metformin use and MBD have found no difference in percent density among patients with diabetes treated with “pills” compared to no treatment, or insulin, [25] an inverse association between diabetes and “mixed/dense breasts” controlled by oral antidiabetic agents compared to diet alone [24], and 5.7% lower percent density for women taking metformin that was attenuated after adjusting for BMI [26]. Our findings add to existing work by using finer measures of exposure (metformin use specifically rather than combining diabetes medications) and outcome (continuous density measures), by adjusting for insulin use—thought to be associated with increased MBD [24]—and by considering co-medications with statin. When considering co-medications, only 33 participants had taken metformin and not statin, which may have limited our ability to detect small associations with MBD for this group, so larger studies examining co-medication are necessary.

We observed consistent associations of at least 5% lower percent density with statin use through all of our sensitivity analyses, which is likely clinically significant as it approaches the magnitude of change associated with reduced risk of breast cancer following tamoxifen use (typically at least 8–10% lower percent density) [40, 41]. Compared to metformin, there has been greater attention evaluating statin use and percent density, and their potential to impact MBD. Lipophilic statins are associated with increased affinity to extra-hepatic tissues and are hypothesized to have differential effects on cancer development than hydrophilic statins [5, 23]. Three small trials of the lipophilic statins simvastatin, lovastatin, or atorvastatin found no change in continuous density measures after 6 months to 1 year of use among fewer than 50 women in midlife who are at high risk for breast cancer [28, 29, 31]. Our cohort contains women across the spectrum of breast cancer risk but it included a higher prevalence of women with cardiometabolic risk factors as compared to the general US population and the women in these trials, though statin lipophilicity was not available for our statin users. An additional large retrospective cohort found no change in BI-RADS over 1–2 years following 1–2 years of statin use according to pharmacy records, though continuous MBD measures, which can capture smaller effects than large BI-RAD categories changes, were not available [27]. Finally, a large Swedish retrospective cohort found decreased percent dense volume and increased non-dense volume among women who had used statin in the prior year [30]. Our findings are supported by this latter study while building on existing work as our population is at greater metabolic risk and therefore has a higher prevalence of statin use compared to the general Swedish population (8.1% of that study population had used statins vs. 27% in our study). Furthermore, we similarly found consistent associations between statin use and dense area, which is a biologically significant measure of density for breast cancer risk [35]. Though prior studies on statin use and MBD are limited and mixed, high blood cholesterol diagnoses (agnostic of medication use) have been inversely associated with percent density and dense area [42]. Furthermore, low blood high-density lipoprotein has been associated with greater dense area [32, 43] or percent density [44, 45], with some exceptions [46]. Together, these findings lend support for the hypothesis that disruption in the biological pathway involving cholesterol synthesis affected both by statin use and by the underlying disease process leading to statin prescription may be associated with MBD.

Although studies have demonstrated high validity of self-reported medication use and duration compared to pharmacy records, particularly for statins [47], our study is still limited by its cross-sectional approach and self-reported medication use, so our findings warrant exploration of this question using longitudinal prospective and clinical data. Although we observed consistency of our findings using PS and IPTW sensitivity analyses, which considered self-reported data on the use of other relevant medication (aspirin, anti-hypertensive medication) and disorders (diabetes, only in the PS for statin use), we remain unable to distinguish the effect of the underlying disease process culminating in the use of these medications from the effects of the medication itself on MBD as medication use nearly completely correlated with disease status (for example, only 8.7% of women who had used neither medication reported having diabetes), our comparison group likely contains both diseased non-medication users with underlying disease and disease-free women, and data were unavailable for clinical measures and diagnosis timing. The predominantly immigrant, majority Hispanic composition of our study population may hinder generalizability of our findings, yet it provides new data in population groups that have been underrepresented in prior studies in this area. Our study is strengthened by the use of reliable, semi-automated, continuous MBD measures in addition to clinical BI-RADS, and a population with a high cardiometabolic burden [32] and therefore high prevalence of metformin and statin use. Thus, we were well-powered to analyze this question compared to previous studies in populations with lower cardiometabolic risk that observed mixed results. Future work should consider this question prospectively, while considering dose, duration, statin lipophilicity, and blood cholesterol, particularly in cohorts with a high prevalence of medication use, such as this one.

## Conclusion

Statin use was consistently associated with lower mammographic breast density, measured both through clinical radiologist assessment and continuous relative and absolute measures, including dense area, a biologically significant indicator of breast cancer risk. Metformin use was associated with lower percent density and higher non-dense area on the mammogram, but this association may be driven by co-medication with statins. Our overall results support that statins may lower mammographic density, but they need confirmation through prospective studies with data on underlying clinical conditions.

## Supplementary information

**Additional file 1 : Table S1.** Model estimates for high density BI-RADS and continuous density measures with medication use. **Table S2.** Characteristics by statin and metformin co-medication. **Table S3.** PS and IPTW sensitivity analyses for high density BI-RADS and continuous density measures with medication use. **Figure S1.** Parameter estimates and 95% confidence intervals for percent density (A), cm^2^ dense area (B), cm^2^ non-dense area (C) with statin (left panel) and metformin (right panel) use in separate fully adjusted models by BMI strata (< 25, 25 to < 30, 30 to < 35, ≥ 35). All models adjust for age, within-strata continuous BMI, education, race, menopausal status, age at first live birth, and insulin use.

## Data Availability

Data are available within the articles and supplementary materials. Additional data may be available on request from the authors.

## Abbreviations

MBD: Mammographic breast density
PD: Percent density
DA: Dense area
NDA: Non-dense area
PS: Propensity score
IPTW: Inverse probability of treatment weighting
BI-RADS: Breast Imaging Reporting and Data System
