# Supplementary material for: Independent and joint cross-sectional associations of statin and metformin use with mammographic breast density
Source: Breast Cancer Res. 2020 Sep 15;22:99. doi: 10.1186/s13058-020-01336-0 (PMC7493153; doi:10.1186/s13058-020-01336-0)
Supplement: Supplementary file 1 — Additional file 1 : Table S1. Model estimates for high density BI-RADS and continuous density measures with medication use. Table S2. Characteristics by statin and metformin co-medication. Table S3. PS and IPTW sensitivity analyses for high density BI-RADS and continuous density measures with medication use. Figure S1. Parameter estimates and 95% confidence intervals for percent density (A), cm2 dense area (B), cm2 non-dense area (C) with statin (left panel) and metformin (right panel) use in separate fully adjusted models by BMI strata (< 25, 25 to < 30, 30 to < 35, ≥ 35). All models adjust for age, within-strata continuous BMI, education, race, menopausal status, age at first live birth, and insulin use. [file 13058_2020_1336_MOESM1_ESM.docx]

# [Supplemental Table 1](#SuppT1)

| **Supplemental Table 1. Model estimates for high density BI-RADS and continuous density measures with medication use.** | | | | | | | | | | | |  |
| --- | --- | --- | --- | --- | --- | --- | --- | --- | --- | --- | --- | --- |
|  | **High density BI-RADS*** | | | **Percent density** | | | **Dense area (cm^2^)** | | | **Non-dense area (cm^2^)** | | |
|  | **RR** | **95% CI** | **p-value** | **β** | **95% CI** | **p-value** | **β** | **95% CI** | **p-value** | **β** | **95% CI** | **p-value** |
| **Model 1 - Sociodemographic factor adjusted: age, education, race** | | | | | |  |  |  |  |  |  |  |
| **Statin use** | 0.55 | (0.41, 0.74) | <0.0001 | -7.26 | (-9.96, -4.56) | <0.0001 | -8.79 | (-14.59, -2.98) | 0.003 | 28.25 | (16.34, 40.15) | <0.0001 |
| **Metformin use** | 0.67 | (0.46, 0.99) | 0.04 | -6.66 | (-10.1, -3.2) | 0.0002 | -2.38 | (-9.73, 4.98) | 0.53 | 32.04 | (17.01, 47.06) | <0.0001 |
| **Model 2 – Sociodemographic factors and BMI adjusted** | | | | |  |  |  |  |  |  |  |  |
| **Statin use** | 0.59 | (0.44, 0.78) | 0.0003 | -6.23 | (-8.67, -3.79) | <0.0001 | -9.85 | (-15.54, -4.16) | 0.0007 | 22.0 | (12.47, 31.54) | <0.0001 |
| **Metformin use** | 0.82 | (0.57, 1.19) | 0.26 | -3.74 | (-6.88, -0.60) | 0.02 | -5.42 | (-12.69, 1.85) | 0.14 | 14.26 | (2.04, 26.48) | 0.02 |
| **Model 3 - Full covariates**† | | | |  |  |  |  |  |  |  |  |  |
| **Statin use** | 0.60 | (0.45, 0.8) | 0.0005 | -6.56 | (-9.05, -4.06) | <0.0001 | -9.05 | (-14.89, -3.22) | 0.002 | 25.21 | (15.47, 34.95) | <0.0001 |
| **Metformin use** | 0.84 | (0.57, 1.23) | 0.37 | -4.20 | (-7.42, -0.99) | 0.01 | -5.08 | (-12.53, 2.38) | 0.18 | 17.48 | (4.95, 30) | 0.006 |
| **Model 4 - Fully and mutually adjusted**† | | | |  |  |  |  |  |  |  |  |  |
| **Statin use** | 0.59 | (0.44, 0.8) | 0.0005 | -6.11 | (-8.71, -3.51) | <0.0001 | -8.61 | (-14.69, -2.53) | 0.006 | 23.18 | (13.03, 33.32) | <0.0001 |
| **Metformin use** | 1.01 | (0.69, 1.48) | 0.96 | -2.03 | (-5.34, 1.28) | 0.23 | -2.01 | (-9.74, 5.72) | 0.61 | 9.23 | (-3.66, 22.11) | 0.16 |
| **Co-medication categories** | | |  |  |  |  |  |  |  |  |  |  |
| **Model 5 - Sociodemographic factor adjusted: age, education, race** | | | | | |  |  |  |  |  |  |  |
| **Statin and**  **metformin** | 0.59 | (0.36, 0.95) | 0.03 | -8.78 | (-12.94, -4.62) | <0.0001 | -6.29 | (-15.28, 2.69) | 0.17 | 39.31 | (21.02, 57.6) | <0.0001 |
| **Metformin alone** | 0.59 | (0.32, 1.09) | 0.09 | -8.42 | (-14.04, -2.80) | 0.003 | -2.84 | (-14.97, 9.29) | 0.65 | 39.37 | (14.67, 64.06) | 0.002 |
| **Statin alone** | 0.51 | (0.36, 0.72) | 0.0002 | -7.38 | (-10.47, -4.3) | <0.0001 | -10.2 | (-16.87, -3.56) | 0.003 | 27.00 | (13.45, 40.55) | <0.0001 |
| **Neither used** | ref. |  |  | ref. |  |  | ref. |  |  | ref. |  |  |
| **Model 6 -Sociodemographic factors and BMI adjusted** | | | | |  |  |  |  |  |  |  |  |
| **Statin and**  **metformin** | 0.69 | (0.43, 1.11) | 0.12 | -6.26 | (-10.04, -2.48) | 0.001 | -9.00 | (-17.83, -0.17) | 0.046 | 23.90 | (9.11, 38.69) | 0.002 |
| **Metformin alone** | 0.78 | (0.44, 1.39) | 0.40 | -4.21 | (-9.33, 0.91) | 0.11 | -7.36 | (-19.32, 4.59) | 0.23 | 13.64 | (-6.38, 33.66) | 0.2 |
| **Statin alone** | 0.54 | (0.38, 0.75) | 0.0003 | -6.65 | (-9.44, -3.87) | <0.0001 | -11.0 | (-17.51, -4.49) | 0.001 | 22.54 | (11.64, 33.45) | <0.0001 |
| **Neither used** | ref. |  |  | ref. |  |  | ref. |  |  | ref. |  |  |
| **Model 7 - Fully adjusted**† | | | |  |  |  |  |  |  |  |  |  |
| **Statin and**  **metformin** | 0.71 | (0.43, 1.16) | 0.17 | -7.07 | (-10.97, -3.17) | 0.0004 | -8.30 | (-17.42, 0.81) | 0.07 | 29.76 | (14.55, 44.96) | 0.0001 |
| **Metformin alone** | 0.77 | (0.44, 1.37) | 0.38 | -4.78 | (-9.92, 0.36) | 0.07 | -7.98 | (-20, 4.04) | 0.19 | 16.04 | (-4.01, 36.09) | 0.12 |
| **Statin alone** | 0.54 | (0.39, 0.76) | 0.0005 | -6.86 | (-9.67, -4.05) | <0.0001 | -10.3 | (-16.83, -3.66) | 0.002 | 25.05 | (14.06, 36.03) | <0.0001 |
| **Neither used** | ref. |  |  | ref. |  |  | ref. |  |  | ref. |  |  |
| † Adjusted for age, BMI, education, race, menopausal status, age at first live birth, and insulin, plus statin and/or metformin use. | | | | | | | | | | | |  |

# [Supplemental Table 2](#SuppT2)

| **SUPPLEMENTAL Table 2. Characteristics by statin and metformin co-medication** | | | | | | | |  |  |  |  |
| --- | --- | --- | --- | --- | --- | --- | --- | --- | --- | --- | --- |
|  |  |  |  |  | | | |  |  |  |  |
|  |  | **Total** | | **Statin and metformin ever** | | **Metformin ever, not statin** | | **Statin ever, not metformin** | | **Neither statin or metformin** | |
|  |  | **n** | **%** | **n** | **%** | **n** | **%** | **n** | **%** | **n** | **%** |
| Total (row percent) | | 770 | 100.0% | 69 | 9.0% | 33 | 4.29% | 140 | 18.2% | 528 | 68.6% |
| **Medication use** | |  |  |  |  |  |  |  |  |  |  |
|  | Metformin ever | 102 | 13.25% | 69 | 100.00% | 33 | 100.00% | 0 | 0.00% | 0 | 0.00% |
|  | Statin ever | 209 | 27.14% | 69 | 100.00% | 0 | 0.00% | 140 | 100.00% | 0 | 0.00% |
|  | Insulin ever | 31 | 4.03% | 12 | 17.39% | 4 | 12.12% | 8 | 5.71% | 7 | 1.33% |
|  | Hormone Replacement Therapy ever | 32 | 4.16% | 3 | 4.35% | 2 | 6.06% | 7 | 5.00% | 20 | 3.79% |
|  | Aspirin ever | 159 | 20.65% | 36 | 52.17% | 9 | 27.27% | 46 | 32.86% | 68 | 12.88% |
|  | Anti-hypertension medications ever | 294 | 38.18% | 51 | 73.91% | 18 | 54.55% | 82 | 58.57% | 143 | 27.08% |
| **Demographic and medical factors** | |  |  |  |  |  |  |  |  |  |  |
| Age | 40 - <45 | 107 | 13.90% | 0 | 0.00% | 3 | 9.09% | 8 | 5.71% | 96 | 18.18% |
|  | 45 - <50 | 170 | 22.08% | 5 | 7.25% | 6 | 18.18% | 17 | 12.14% | 142 | 26.89% |
|  | 50 - <55 | 218 | 28.31% | 17 | 24.64% | 16 | 48.48% | 31 | 22.14% | 154 | 29.17% |
|  | 55 or older | 275 | 35.71% | 47 | 68.12% | 8 | 24.24% | 84 | 60.00% | 136 | 25.76% |
|  | Mean [SD]  (min - max) | 51.96 [5.63]  (40.04-61.02) | | 55.98 [3.66]  (46.10-60.92) | | 51.85 [4.81] (42.13-59.67) | | 54.69 [5.01] (40.76-61.02) | | 50.71 [5.57] (40.04-61.02) | |
| Education | High School or less | 359 | 46.62% | 48 | 69.57% | 15 | 45.45% | 66 | 47.14% | 230 | 43.56% |
|  | Some college | 179 | 23.25% | 11 | 15.94% | 9 | 27.27% | 37 | 26.43% | 122 | 23.11% |
|  | Bachelor's or higher | 232 | 30.13% | 10 | 14.49% | 9 | 27.27% | 37 | 26.43% | 176 | 33.33% |
| Race | Hispanic | 604 | 78.44% | 58 | 84.06% | 23 | 69.70% | 118 | 84.29% | 405 | 76.70% |
|  | Non-Hispanic white | 60 | 7.79% | 2 | 2.90% | 0 | 0.00% | 6 | 4.29% | 52 | 9.85% |
|  | Non-Hispanic black | 88 | 11.43% | 7 | 10.14% | 9 | 27.27% | 13 | 9.29% | 59 | 11.17% |
|  | Non-Hispanic, Asian or other race | 18 | 2.34% | 2 | 2.90% | 1 | 3.03% | 3 | 2.14% | 12 | 2.27% |
| Diabetes | Any | 160 | 20.78% | 66 | 95.65% | 28 | 84.85% | 20 | 14.29% | 46 | 8.71% |
|  | Type 1 | 12 | 1.56% | 6 | 8.70% | 3 | 9.09% | 2 | 1.42% | 1 | 0.19% |
|  | Type 2 | 158 | 20.52% | 58 | 84.06% | 24 | 72.73% | 10 | 7.14% | 8 | 1.52% |
|  | Gestational | 72 | 9.35% | 16 | 23.19% | 7 | 21.21% | 11 | 7.86% | 38 | 7.20% |
| BMI | 18.5 - < 25 | 134 | 17.40% | 8 | 11.59% | 3 | 9.09% | 18 | 12.86% | 105 | 19.89% |
|  | 25 - < 30 | 247 | 32.08% | 17 | 24.64% | 2 | 6.06% | 45 | 32.14% | 183 | 34.66% |
|  | 30 - < 35 | 215 | 27.92% | 22 | 31.88% | 15 | 45.45% | 43 | 30.71% | 135 | 35.57% |
|  | ≥ 35 | 174 | 22.60% | 22 | 31.88% | 13 | 39.39% | 24 | 24.29% | 105 | 19.89%% |
|  | Mean [SD]  (min - max) | 30.91 [6.56]  (16.37-70.80) | | 33.12 [7.02] (21.33-59.06) | | 34.82 [5.75] (20.96-45.88) | | 31.15 [5.79] (21.26-53.38) | | 30.31 [6.61]  (16.37-70.80) | |
| **Reproductive history** | |  |  |  |  |  |  |  |  |  |  |
| Age at menarche | <12 | 204 | 26.49% | 15 | 21.74% | 11 | 33.33% | 30 | 21.43% | 148 | 28.03% |
|  | 12 | 138 | 17.92% | 14 | 20.29% | 6 | 18.18% | 23 | 16.43% | 95 | 17.99% |
|  | 13 | 167 | 21.69% | 18 | 26.09% | 8 | 24.24% | 34 | 24.29% | 107 | 20.27% |
|  | > 14 | 258 | 33.51% | 21 | 30.43% | 8 | 24.24% | 52 | 37.14% | 177 | 33.52% |
|  | Missing | 3 | 0.39% | 1 | 1.45% | 0 | 0.00% | 1 | 0.71% | 1 | 0.19% |
| Parity | Nulliparous | 101 | 13.12% | 7 | 10.14% | 4 | 12.12% | 20 | 14.29% | 70 | 13.26% |
|  | 1-2 children | 368 | 47.79% | 34 | 49.28% | 14 | 42.42% | 65 | 46.43% | 255 | 48.30% |
|  | 3 or more children | 301 | 39.09% | 28 | 40.58% | 15 | 45.45% | 55 | 39.29% | 203 | 38.45% |
| Age at first live birth | Nulliparous | 101 | 13.12% | 7 | 10.14% | 4 | 12.12% | 20 | 14.29% | 70 | 13.26% |
|  | <25 years old | 400 | 51.95% | 43 | 62.32% | 20 | 60.61% | 71 | 50.71% | 266 | 50.38% |
|  | 25 - <35 years old | 212 | 27.53% | 15 | 21.74% | 8 | 24.24% | 40 | 28.57% | 149 | 28.22% |
|  | >35 years old | 57 | 7.40% | 4 | 5.80% | 1 | 3.03% | 9 | 6.43% | 43 | 8.14% |
|  | Mean [SD]  (min - max) | 24.18 [6.19]  (13.0-42.0) | | 23.13 [6.05] (13.0-39.0) | | 22.22 [5.76] (15.0-37.0) | | 24.05 [5.60] (15.0-42.0) | | 24.48 [6.39] (13.0-42.0) | |
| **Breast cancer risk factors** | |  |  |  |  |  |  |  |  |  |  |
| Post-menopause | | 455 | 59.09% | 62 | 89.86% | 16 | 48.48% | 117 | 83.57% | 260 | 49.24% |
| First degree family history of breast cancer | | 95 | 12.34% | 11 | 15.94% | 4 | 12.12% | 12 | 8.57% | 68 | 12.88% |
| Age at menopause: Mean [SD] (min-max) | | 46.39 [6.30]  (19.40-58.00) | | 45.38 [6.97] (26.6-57.6) | | 46.52 [6.82]  (30.10-52.70) | | 45.97 [6.43] (19.40-58.00) | | 46.80 [6.05] (22.60-57.20) | |
| Missing were excluded. For coronary heart disease, 95 participants did not answer this question. | | | | | | | | | | | |

# [Supplemental Table 3](#SuppT2)

| **Supplemental Table 3. PS and IPTW sensitivity analyses for high density BI-RADS and continuous density measures with medication use.** | | | | | | | | | | | | | |
| --- | --- | --- | --- | --- | --- | --- | --- | --- | --- | --- | --- | --- | --- |
|  | **High Risk BI-RADS** | | | **% Density** | | | | **Dense Area (cm2)** | | | **Non-Dense Area** | | |
|  | **RR** | **95% CI** | **p-value** | **β** | **95% CI** | **p-value** | | **β** | **95% CI** | **p-value** | **β** | **95% CI** | **p-value** |
| **Model 1 - PS Adjusted** | | | | | | | | | | | | | |
| **Statin Use** | 0.66 | (0.48, 0.91) | 0.01 | -4.96 | (-8.1, -1.8) | | 0.002 | -7.3 | (-14.0, -0.6) | 0.03 | 18.4 | (4.5, 32.3) | 0.0096 |
| **Metformin Use** | 0.96 | (0.66, 1.41) | 0.85 | -3.8 | (-7.5, -0.1) | | <0.05 | -3.4 | (-11.4, 4.6) | 0.41 | 14.6 | (-1.3, 30.5) | 0.07 |
| **Model 2 - Statin PS and adjusted for metformin** | | | | | | | | | | | | | |
| **Statin Use** | 0.66 | (0.48, 0.91) | 0.01 | -4.96 | (-8.1, -1.8) | | 0.002 | -7.4 | (-14.1, -0.7) | 0.03 | 18.1 | (4.2, 31.9) | 0.0108 |
| **Metformin Use** | 1.19 | (0.76, 1.85) | 0.44 | 0.16 | (-4.1, 4.4) | | 0.94 | 7.9 | (-1.2, 17.0) | 0.09 | 17.4 | (-1.5, 36.2) | 0.07 |
| **Model 3 - Metformin PS and adjusted for statin** | | | | | | | | | | | | | |
| **Statin Use** | 0.66 | (0.49, 0.89) | 0.007 | -6.1 | (-9.0, -3.2) | | <0.0001 | -12.6 | (-18.8, -6.4) | 0.95 | 12.3 | (-0.2, 24.8) | 0.05 |
| **Metformin Use** | 1.08 | (0.73, 1.59) | 0.70 | -2.03 | (-5.8, 1.8) | | 0.29 | 0.3 | (-7.9, 8.4) | 0.95 | 11.0 | (-5.3, 27.3) | 0.19 |
| **Model 1 - IPTW** | | | | | | | | | | | | | |
| **Statin Use** | 0.58 | (0.37, 0.92) | 0.02 | -5.65 | (-8.1, -3.2) | | <0.0001 | -11.3 | (-15.7, -6.8) | <0.0001 | 22.4 | (10.0, 34.9) | 0.0004 |
| **Metformin Use** | 0.72 | (0.44, 1.17) | 0.19 | -5.32 | (-7.7, -2.9) | | <0.0001 | -2.0 | (-6.8, 2.8) | 0.42 | 21.3 | (10.8, 31.8) | <0.0001 |
| **Model 2 - Statin IPTW and adjusted for metformin** | | | | | | | | | | | | | |
| **Statin Use** | 0.58 | (0.37, 0.92) | 0.02 | -5.57 | (-8.0, -3.2) | | <0.0001 | -11.3 | (-15.7, -6.8) | <0.0001 | 22.1 | (9.8, 34.4) | 0.0004 |
| **Metformin Use** | 0.65 | (0.4, 1.06) | 0.09 | -9.09 | (-12.8, -5.4) | | <0.0001 | 0.2 | (-6.7, 7.2) | 0.95 | 42.3 | (23.1, 61.4) | <0.0001 |
| **Model 3 - Metformin IPTW and adjusted for statin** | | | | | | | | | | | | | |
| **Statin Use** | 0.65 | (0.38, 1.12) | 0.12 | -8.73 | (-11.3, -6.2) | | <0.0001 | -14.5 | (-19.6, -9.4) | <0.0001 | 23.9 | (12.7, 35.1) | <0.0001 |
| **Metformin Use** | 0.82 | (0.5, 1.34) | 0.42 | -2.7 | (-5.2, -0.3) | | 0.03 | 2.4 | (-2.6, 7.3) | 0.35 | 14.1 | (3.2, 25.0) | 0.01 |
| *Sample size for statin as a primary treatment =762; n=764 for metformin. | | | | | | | | | | | | | |
| ‡ PS = Propensity score; IPTW = Inverse probability of treatment weighting  Propensity scores were modeled using the following variables: diabetes (statin PS score only), benign breast disease, breast biopsy, aspirin use, anti-hypertension medication use, age at menopause, age at menarche, and breast cancer family history. | | | | | | | | | | | | | |

**Supplemental Figure 1.**


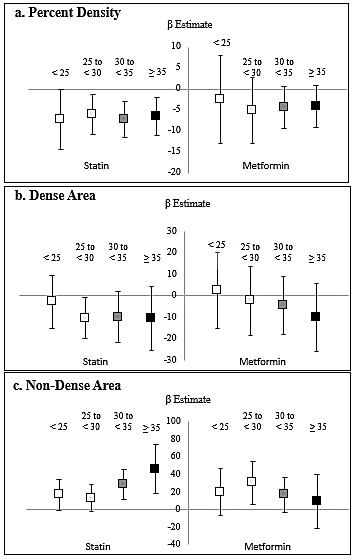


**Supplemental Figure 1 legend:** Parameter estimates and 95% confidence intervals for percent density (A), cm^2^ dense area (B), cm^2^ non-dense area (C) with statin (left panel) and metformin (right panel) use in separate fully adjusted models by BMI strata (< 25, 25 to < 30, 30 to < 35, ≥ 35). All models adjust for age, within-strata continuous BMI, education, race, menopausal status, age at first live birth, and insulin use.
